# Work Environment Characteristics and Teacher Well-Being: The Mediation of Emotion Regulation Strategies

**DOI:** 10.3390/ijerph13090907

**Published:** 2016-09-13

**Authors:** Hongbiao Yin, Shenghua Huang, Wenlan Wang

**Affiliations:** 1 Department of Curriculum and Instruction, Faculty of Education, The Chinese University of Hong Kong, Hong Kong, China; yinhb@cuhk.edu.hk (H.Y.); huang@cuhk.edu.hk (S.H.); 2 Department of Curriculum and Instruction, School of Educational Science, South China Normal University, Guangzhou 510631, China

**Keywords:** emotional job demands, trust in colleagues, emotion regulation, teacher well-being, school environment, mediating effect

## Abstract

Based on an adjusted Job Demands-Resources (JD-R) model that considers the mediation of personal resources, this study examined the relationships between two characteristics of teachers’ work environment (i.e., emotional job demands and trust in colleagues) and two indicators of teachers’ well-being (i.e., teaching satisfaction and emotional exhaustion). In particular, the study focused on how emotion regulation strategies (i.e., reappraisal and suppression) mediate these relationships. Data collected from a questionnaire survey of 1115 primary school teachers in Hong Kong was analyzed to test the hypothesized relationships. The results of structural equation modeling indicated that: (1) the emotional job demands of teaching were detrimental to teacher well-being, whereas trust in colleagues was beneficial; (2) both emotion regulation strategies mediated the relationships between both emotional job demands and trust in colleagues and teacher well-being; and (3) teachers who tend to use more reappraisal may be psychologically healthier than those tend to adopt more suppression. These findings support the applicability of the JD-R model to school settings and highlight the role of teachers’ emotion regulation in teachers’ well-being. Implications for the improvement of school environments and teachers’ well-being are identified.

## 1. Introduction

The Job Demands-Resources (JD-R) model has been widely used to explain the relationships between work environment characteristics and employees’ performance and well-being [1,2]. According to the JD-R model, job characteristics can be divided into two categories: job demands and job resources. These two types of job characteristics are related to employees’ stress and motivation, respectively, and to some organizational outcomes such as performance, turnover intention, and health problems [3,4,5]. Most studies using the JD-R model have been conducted in fields other than education, but recently a few studies have tested the applicability of the JD-R model in school settings (e.g., [6,7,8]).

Teacher emotion in school settings has increasingly attracted the attention of researcher in recent years [9,10,11]. The relevance of emotion to teaching and teachers in both schools [12,13,14,15] and universities [16,17,18] has been widely recognized. Teachers are generally expected to up-regulate pleasant emotions while down-regulate negative or anti-social emotions [9,19]. However, although emotion is well known to closely relate to one’s well-being [20,21,22], no attempt has been made to explore the links among teachers’ emotion regulation, work environment, and their well-being.

Teachers’ emotion regulation is an underexplored issue in the field of educational research, although it has been suggested that teachers’ ability to regulate their emotions is important for their well-being and the effectiveness of classroom management [23,24]. Until very recently, few empirical studies explored the role of emotion regulation in teachers’ work. For example, Sutton’s qualitative study demonstrated that teachers believed that regulating their emotions helped them achieve their teaching goals, and that teachers who were successful at regulating their emotions may be less susceptible to burnout [25]. Sutton and Harper argued that the effectiveness and consequences of emotion regulation are related to the strategies that teachers adopted [26]. In another qualitative study, Yin classified the strategies adopted by teachers to regulate their emotions in the classroom, and suggested that emotion regulation strategies help teachers fulfill their professional goals and may therefore influence their well-being [27]. Brackett, Palomera, Mojsa-Kaja, Reyes, and Salovey’s quantitative study found that teachers’ ability to regulate their emotions is positively associated with their positive affect, job satisfaction, and personal accomplishment, which is one component of burnout [28]. However, little work has been done to systematically examine the relationships between teachers’ emotion regulation and its antecedents and their effects on teaching effectiveness and teacher well-being.

Therefore, the present study examined the relationships between two specific work environment characteristics (i.e., emotional job demands and trust in colleagues), teachers’ emotion regulation strategies (i.e., reappraisal and suppression), and two indicators of teacher well-being (i.e., teaching satisfaction and emotional exhaustion), with a particular focus on the mediating role of emotion regulation. In the following section, the JD-R model and relevant studies are reviewed to establish the hypotheses.

### 1.1. JD-R Model and Teacher Well-Being

In the JD-R model, the two core concepts, job demands and job resources, are broadly defined to include physical, cognitive, and emotional demands and numerous resources located at organizational, interpersonal, work, and task levels. Bakker and Demerouti defined job demands as job characteristics that “require sustained physical and/or psychological (cognitive or emotional) effort or skills and are therefore associated with certain physiological and/or psychological costs,” and job resources as job characteristics that are either “functional in achieving work goals,” or can “reduce job demands and the associated physiological and psychological costs” ([1], p. 312). Therefore, the JD-R model is conceptualized as a dual process model that includes two parallel processes: the health impairment process and the motivational process. In the health impairment process, job demands lead to individual efforts to maintain performance and fatigue after-effects; in the health improvement process, the intrinsic and extrinsic motivational potentials of job resources lead to high work engagement. Job demands and resources may also interact with each other during the two development processes of job stress and job motivation [2].

The JD-R model provides a powerful framework for exploring the relationships between the characteristics of the work environment and employees’ well-being and performance. As stated above, the validity of the JD-R model in different work contexts has been well documented [3,4,29]. In the field of educational or school psychology, a few studies have applied the JD-R model to the exploration of teachers’ well-being or performance. For instance, Simbula found that teachers’ work engagement mediated the effect of co-workers’ support on job satisfaction and mental health, whereas their exhaustion mediated the relationship between work/family conflict and job satisfaction and mental health [6]. Hakanen, Bakker, and Schaufeli found that job demands and job resources related to teachers’ burnout and engagement in the expected ways, which further influenced their health and organizational commitment [7]. Bakker and Bal also found that teachers’ week-levels of autonomy, exchange with their supervisor, and opportunities for development were positively related to weekly engagement, which, in turn, was positively related to weekly performance [8].

In this study, particular attention was paid to the emotional side of teaching and the role of emotional regulation, thus, the emotional job demands of teaching, rather than its physical or cognitive demands, was chosen as the job demand under the JD-R framework. The emotional job demands of teaching emerge from teachers’ interactions with students, parents, and colleagues. These emotional job demands denote the specific requirements of the teaching profession on teachers’ emotional expressions, such as showing positive emotions while suppressing negative ones [30]. Meanwhile, teachers’ perception of trust in colleagues was chosen correspondingly as a work environment resource rather than other job resources such as teacher autonomy or social support, because trust plays a more fundamental role in social interactions. Level of trust in colleagues indicates teachers’ willingness to be vulnerable to their colleagues, based on their belief that colleagues are benevolent, reliable, competent, honest, and open [31]. Among other resources possessed by teachers, trust is the “foundation of school effectiveness”, without which real supportive environment cannot be created and real job autonomy cannot be delivered [32]. However, trust in colleagues is often thought to be the basis of “taken-for-granted” aspects of social interaction and deserves more attention.

Emotional exhaustion and teaching satisfaction were used as indicators of teacher well-being in this study. Emotional exhaustion is a state of fatigue that emerges when one’s emotional resources are used up [33]. It is a proximal variable of burnout, which is a long-term result of some interpersonal interactions. Teaching satisfaction represents the job satisfaction of teachers, which is an important aspect of their life satisfaction [34]. Job satisfaction has been demonstrated to be closely related to individuals’ psychological well-being and represents a desirable part-whole relationship [35,36]. Emotional exhaustion and job satisfaction have been used in many previous studies as indicators of subjective well-being [35,37,38,39,40].

Although there is still a scarcity of research, some studies have provided preliminary results about the effects of emotional job demands and trust in colleagues on teachers’ well-being. For example, Näring et al. found that the emotional job demands of teaching positively predict teachers’ sense of emotional exhaustion 39 [28], a finding that is consistent with those in health care research (e.g., [41,42]). In addition, Wang, Yin, and Huang’s study of service workers found that emotional job demands significantly increased employees’ emotional exhaustion and reduced their job satisfaction [43]. In contrast, trust in colleagues has often been found to be associated with teachers’ positive well-being indicators or other motivational attributes, such as job satisfaction [44], personal efficacy [45], and commitment to students [46]. Moreover, Van Maele and Van Houtte recently found that trust in schools can act as a buffer against teacher burnout [47].

Thus, we hypothesize the following:

H1: The emotional job demands of teaching are positively related to teachers’ emotional exhaustion (H1a), but negatively related to their teaching satisfaction (H1b).

H2: Trust in colleagues is negatively related to teachers’ emotional exhaustion (H2a), but positively related to their teaching satisfaction (H2b).

### 1.2. Teachers’ Emotional Regulation as a Mediating Process

Although the JD-R model is helpful in explaining the relationships between characteristics of the work environment and employees’ well-being and performance, it has recently been criticized for overlooking “personal resources”, because most psychological approaches assume that human behavior results from the interaction between environmental and personal factors [48]. These personal resources denote the psychological characteristics that are related to individuals’ ability to successfully control and affect their environment. These personal resources mediate the relationships between job characteristics and well-being [2]. For instance, researchers have found that individuals’ satisfaction of basic psychological needs (i.e., competence, autonomy, and belongingness) mediated the relationships between job demands and exhaustion, between job resources and vigor, and between job resources and exhaustion [49].

The stress and coping theory developed by Lazarus and his colleagues [50,51] also lends support to the importance of personal coping strategies as mediators. Lazarus and Folkman suggested that the stress management process has three stages: the cognitive (stress) appraisal, the coping process, and the adaptational outcomes, in which the coping strategies adopted by individuals mediate the relationships between the stressful conditions and individuals’ adaptation [51]. Therefore, the JD-R model should be expanded to include emotion regulation as part of the mediating process.

Gross suggested that emotions arise automatically or with conscious awareness when external stimuli cause important things to be at stake; the process of influencing one’s own emotions is called emotion regulation [52]. Teachers’ emotion regulation reflects their ability to successfully interact with their work environment and influence their emotions in workplace. As a function of the interaction between environmental and personal factors, emotion regulation strategies adopted by teachers may further influence their well-being. Gross identified two broad types of emotion regulation strategies: cognitive reappraisal and expressive suppression [20]. Reappraisal is an antecedent-focused emotion regulation that involves “construing a potentially emotional-eliciting situation in nonemotional terms,” whereas suppression is a response-focused emotion regulation that involves “inhibiting ongoing emotional expressive behavior” ([20], p. 283).

#### 1.2.1. Emotional Job Demands, Trust in Colleagues, and Teachers’ Emotional Regulation

Schools and classrooms are complex emotional arenas, and teachers are constantly exposed to the emotional demands placed on them. According to the stress and coping theory, the emotional job demands of teaching, which are rooted in the social expectations and professional norms of teaching [30], may serve as external stimuli that triggers teachers’ appraising situation as stressful and adopting subsequent coping strategies. However, other environmental and personal factors may influence teachers’ adoption of certain type of emotional regulation strategies (i.e., coping strategies) [52]. Some tend to reappraise the situation while others tend to suppress their emotional expression. It is also possible that teachers may suppress their emotional expressions and try to reappraise the situation simultaneously during class. Empirically, Lo’s study of nursing students empirically demonstrated that the stress resulting from the emotional job demands of nursing correlated with the nurses’ avoidance coping and negative self-esteem [53]. Peng, Wong, and Che found that the emotional demands of a job increased employees’ use of coping strategies [54]. Given the characteristics of teachers’ work environment, it is credible to suppose that the emotional job demands of teaching could impel teachers to use either reappraisal or suppression strategies.

Thus, we hypothesize the following:

H3: The emotional job demands of teaching are positively related to reappraisal (H3a) and suppression (H3b).

As an element of a constructive school environment, trust in colleagues also plays important roles in teachers’ emotion regulation processes. People may feel more comfortable being themselves when safety has been ensured [55]. Therefore, teachers in a trustful environment may evaluate their work as less stressful and more relaxed, thus, needing no coping strategy. In addition, teachers who trust their colleagues tend to be more authentic, and thus reduce the use of reappraisal or suppression strategies. Therefore, it is credible to assume that trust in colleagues may decrease teachers’ use of both reappraisal and suppression strategies, even though relevant empirical results are rare.

Thus, we hypothesize the following:

H4: Trust in colleagues is negatively related to reappraisal (H4a) and suppression (H4b).

#### 1.2.2. Emotion Regulation and Teacher Well-Being

Gross classified the consequences of emotion regulation into three types: affective, cognitive, and social consequences [20]. Lazarus particularly suggested that coping is capable of mediating the emotional outcomes from the beginning to the end of an encounter [50]. The relationship between emotion regulation and teacher well-being still needs to be elaborated.

King and Emmons proposed that a lack of emotional expression combined with a desire to express it may be detrimental to an individual’s health [56]. Response-focused regulation (i.e., suppression) may be harmful because individuals cannot express emotions that have already formed. In contrast, antecedent-focused regulation (i.e., reappraisal) may be helpful because it allows individuals to use some cognitive techniques to form the required emotions. Therefore, some researchers have argued that reappraisal may be more effective than suppression for teachers trying to manage their emotions in classroom settings [23,27].

Recently, empirical studies have also explored the consequences of emotion regulation in different contexts, including its effects on individuals’ well-being. Brotheridge and Lee found that suppression appears to be associated with prolonged effort, which is in turn linked to adverse health and well-being [57]. Gross and John found that using reappraisal is associated with better interpersonal functioning and positively related to well-being, whereas using suppression is associated with worse interpersonal functioning and negatively related to well-being [21]. Researchers also found that for school teachers, suppression is negatively related to job satisfaction but positively related to emotional exhaustion, whereas reappraisal is positively related to job satisfaction but negatively related to negative affect [58].

In the field of education research, Chang suggested that teachers’ burnout may come from individual factors, organizational factors, and their interactions (transactional factors) and that the habitual pattern in teachers’ judgments (appraise) about student behavior is an important transactional factor that contribute to teachers’ unpleasant emotion and burnout [59]. A integrative model has been described in her recently work which proposed that teachers’ appraisals of disruptive behavior may lead to episodic unpleasant emotions, which could further be handled by using different coping strategies including cognitive reappraisal and expressive suppression [60]. Results indicated that the reappraisal strategies were negative while the suppression strategies were positive related to burnout [60].

Therefore, it is credible to assume that teachers who habitually use more reappraise strategies may be more satisfied with their work and less likely to be emotionally exhausted, whereas teachers who habitually suppress emotions may be at higher risk of experiencing emotional exhaustion and feel less satisfied with their work.

Thus, we hypothesize the following:

H5: Reappraisal is negatively related to emotional exhaustion (H5a) and positively related to teaching satisfaction (H5b).

H6: Suppression is positively related to emotional exhaustion (H6a) and negatively related to teaching satisfaction (H6b).

**Figure 1:**
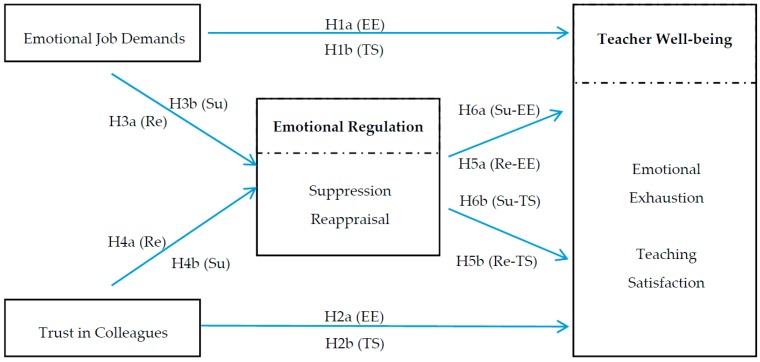
The hypothesized model. *Note*: EE = emotional exhaustion, TS = teaching satisfaction, Su = Suppression, Re = Reappraisal.

In short, this study integrated emotion regulation strategies into the JD-R model as mediating processes. Specifically, using a sample of Hong Kong primary school teachers, we examined (a) the relationships between two characteristics of teachers’ work environment, i.e., emotional job demands of teaching and trust in colleagues, and two well-being indicators, i.e., emotional exhaustion and teaching satisfaction; and (b) the mediating role of two emotion regulation strategies (i.e., reappraisal and suppression) in the relationships between job characteristics and teacher well-being. The hypothesized model tested in this study is shown in Figure 1.

## 2. Materials and Methods

### 2.1. Participants

A questionnaire survey was conducted for data collection in the November 2015 to January 2016 period. Sixty primary schools in Hong Kong were invited to participate in the survey by means of an envelope enclosing an introduction letter and 30 to 70 copies of the questionnaire (depending on the number of teaching staff in the school). The introduction letter clearly stated the nature, purpose, and procedure of the study. The participants took part in this survey on a voluntary basis and were not required to provide a written consent form.

A total of 2500 copies of questionnaires were distributed, and the final sample consisted of 1115 teachers from 38 primary schools in Hong Kong, making a response rate at 44.6%. The sample comprised 252 males (22.6%), 852 females (76.1%), and 11 participants (1.0%) who did not report their gender. The participants had an average of 15 years of work experience.

### 2.2. Measures

#### 2.2.1. Emotional Job Demands of Teaching and Trust among Colleagues

The emotional job demands were measured by the Emotional Job Demands of Teaching Scale developed by Yin [61]. This is a unidimensional scale consisting of four items that mainly assess teachers’ perceptions of the emotional demands of teaching. Examples of these items include “To perform my teaching well, I have to spend most of my time interacting with others (e.g., students, parents and colleagues)” and “I have to use my emotions and behavior to create a reassuring climate for my students and their parents.” The construct validity of this scale had been confirmed in previous research (Chi-square (*χ*^2^) = 8.21, Degrees of freedom (*df*) = 2, *p* = 0.02, Root Mean Square Error of Approximation (RMSEA) = 0.056, Comparative Fit Index (CFI) = 0.98, Tucker-Lewis index (TLI) = 0.94) [61]. The Cronbach’s alpha (α) of the scale in the present study was 0.69, indicating an acceptable internal consistency.

Trust among colleagues was measured by the Trust in Colleague Scale developed by Yin et al. [45]. This is a unidimensional instrument containing five items that assess the five facets of trust between teachers and their colleagues: benevolence, reliability, honesty, competence, and openness. Examples of these items include “Even in difficult situations, teachers in this school can depend on each other” and “Teachers in this school have faith in the integrity of their colleagues.” The construct validity of this scale had been confirmed in previous research (*χ*^2^ = 7.85, *df* = 5, *p* = 0.17, RMSEA = 0.040, CFI = 0.99, TLI = 0.99) [45]. The Cronbach’s α of the scale in this study was 0.88, indicating a sound internal consistency.

#### 2.2.2. Emotional Regulation Strategies

Teachers’ emotional regulation strategies were measured by the Emotional Regulation Questionnaire (ERQ) developed by Gross and John [21]. The ERQ is a bi-factor, 10-item instrument, of which six items are used to measure reappraisal strategies and four items are used to measure suppression strategies. Examples of the ERQ include “When I want to feel more positive emotions (such as joy or amusement), I change what I’m thinking about” (i.e., reappraisal), and “I control my emotions by not expressing them” (i.e., suppression). The convergent validity and discriminant validity had been confirmed in previous research by calculating the convergent and discriminant relations between emotional regulation strategies (i.e., reappraisal & suppression) and other relevant constructs [21]. In this study, we had adjusted the scale by adding “in teaching” to each items when preparing our Chinese version questionnaires, which helps to make sure that the teachers will respond to the items according to their experiences at school. The Cronbach’s α values in the present study were 0.79 for reappraisal and 0.72 for suppression, indicating an acceptable internal consistency.

#### 2.2.3. Emotional Exhaustion and Teaching Satisfaction

Teachers’ emotional exhaustion was measured by the five items of the Maslach Burnout Inventory—General Survey, which is a commonly used questionnaire developed by Maslach et al. [33]. Examples of these items include “I feel very tired to face the day’s work every morning when I get up” and “I feel exhausted after work.” The convergent validity and discriminant validity had been confirmed in previous research [33]. The Cronbach’s α of the scale in the present study was 0.91, indicating a sound internal consistency.

Teaching satisfaction was measured by the Teaching Satisfaction Scale developed by Ho and Au [62]. This is a single-factor, 5-item scale. Examples of these items include “In most ways, being a teacher is close to my ideal” and “My conditions of being a teacher are excellent.” The convergent validity, criterion-related validity, and incremental validity had been confirmed by Ho and Au [62]. The Cronbach’s α of the scale in the present study was 0.89, indicating a sound internal consistency.

All these scales were administered in Chinese language. For all of the scales mentioned above, the participants were required to rate their responses on a 5-point Likert scale ranging from “1 = strongly disagree” to “5 = strongly agree.”

### 2.3. Statistical Analyses

Structural equation modeling (SEM) was used to examine the relationships between the constructs of interest. SEM can be used to assess unobservable latent constructs defined by one or more observed variables, and to model all of the parameters simultaneously. The statistical program Mplus 7 (Muthén & Muthén, Los Angeles, CA, USA) was used to conduct the analyses.

The whole test process followed the two-step procedure advised by Anderson and Gerbing [63]. That is, the measurement model was first tested to confirm the construct validity, and then the structural model was used to test the hypothesized model. The model fit was assessed using the Chi-square value, CFI, TLI, and RMSEA. The data fit is acceptable when CFI and TLI are no less than 0.90 (the higher, the better), and an acceptable fit requires the RMSEA to be under 0.08 (the lower, the better) [64]. Moreover, given the large sample in this study, a set of Sobel tests [65] was adopted to further confirm the mediation effects.

## 3. Results

### 3.1. Reliability, Descriptive Statistics, and Correlations

The descriptive results, correlations, and reliabilities are presented in Table 1. Teachers scored highest in their perception of emotional job demands (Mean (*M*) = 3.94, Standard Deviation (*SD*) = 0.51) and lowest in their strategies of suppression (*M* = 3.07, *SD* = 0.66). For the correlations, except for the non-significant relationships between emotional job demands and both trust among colleagues and teacher satisfaction, all of the variables moderately correlated with each other.

These correlations followed the definitions of these variables, providing preliminary evidence for the construct validity of the scales.

### 3.2. Measurement Model

Before testing the structural model, the measurement model was tested; confirmatory factor analysis (CFA) was used to examine the construct validity of the six scales. The CFA results revealed an acceptable fit to the data (*χ*^2^ = 1520.77, *df* = 362, *p* < 0.001, RMSEA = 0.054, CFI = 0.92, TLI = 0.91). The factor loadings of all of the latent variables were significant, ranging from 0.55 to 0.89. These results indicated that the construct validity of all of the scales was acceptable, and all of the latent variables were well represented by the indicators.

### 3.3. Structural Model

**Figure 2:**
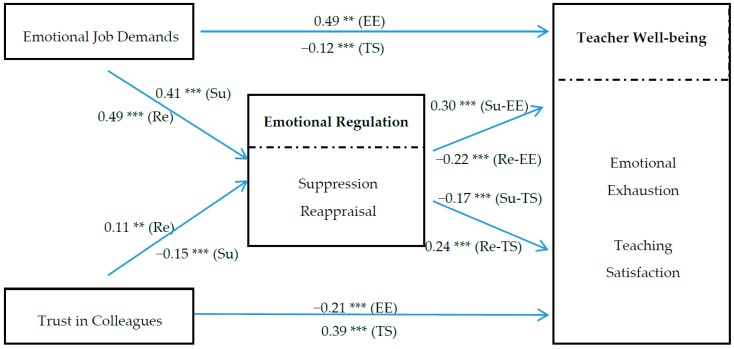
Path analysis results of the hypothesized model. *Note*: ** *p* < 0.01, *** *p* < 0.001. EE = Emotional Exhaustion, TS = Teaching Satisfaction, Su = Suppression, Re = Reappraisal.

Once the construct validity of the measurement model was established, we tested the structural model to examine the direct and indirect relationships between the job characteristics of teaching, teachers’ emotion regulation, and well-being indicators (see Figure 2). Overall, the results shown in Figure 2 indicated an acceptable fit between our theoretical model and the data (*χ*^2^ = 1570.77, *df* = 363, *p* < 0.001, RMSEA = 0.055, CFI = 0.92, and TLI = 0.91).

#### 3.3.1. General Results

The SEM results indicated that emotional job demands were positively associated with emotional exhaustion (Beta coefficient (β) = 0.49, *p* < 0.001) and negatively associated with teaching satisfaction (β = −0.12, *p* < 0.001), supporting H1. In contrast, trust in colleagues was positively related to teaching satisfaction (β = 0.39, *p* < 0.001) and negatively related to emotional exhaustion (β = −0.21, *p* < 0.001), supporting H2.

As expected, significant associations existed between the emotional job demands of teaching and the two emotional regulation strategies (Reappraisal: β = 0.49, *p* < 0.001; Suppression: β = 0.41, *p* < 0.001), supporting H3. However, trust in colleagues was found to be negatively related to suppression (β = −0.15, *p* < 0.001) but positively related to reappraisal (β = 0.11, *p* < 0.01), H4b was supported while H4a was not.

Paths between emotion regulation strategies and teacher well-being were also in accordance with the expected directions. Specifically, reappraisal was positively associated with teaching satisfaction (β = 0.24, *p* < 0.001) and negatively related to emotional exhaustion (β = −0.22, *p* < 0.001); suppression was negatively associated with teaching satisfaction (β = −0.17, *p* < 0.001) and positively related to emotional exhaustion (β = 0.30, *p* < 0.001). H5 and H6 were thus supported.

#### 3.3.2. Testing the Mediation Effects

The SEM results provided some evidence for the mediating effects of emotional regulation strategies. Subsequently, a series of Sobel tests were performed to confirm the significance of the mediation relations. The direct, indirect, and total effects and the Sobel test results are shown in Table 2.

The Sobel tests demonstrated that all eight indirect effects were significantly different from zero, and thus the hypothesized mediation effects were confirmed. Specifically, in addition to their direct and negative association with teaching satisfaction (direct effect = −0.12), emotional job demands were indirectly and positively related to teaching satisfaction via reappraisal (indirect effect = 0.12) and indirectly and negatively associated with teaching satisfaction via suppression (indirect effect = −0.07). As a result, the total effect of emotional job demand on teaching satisfaction was −0.07. The interaction between emotional job demands and emotional exhaustion was exactly opposite to that between emotional job demands and teaching satisfaction: the total effect of emotional job demand on emotional exhaustion was 0.51.

In contrast, trust in colleagues was directly and positively related to teaching satisfaction (direct effect = 0.39). Furthermore, it was indirectly and significantly associated with teaching satisfaction via reappraisal (indirect effect = 0.03) and suppression (indirect effect = 0.03). Trust in colleagues also was directly and negatively related to emotional exhaustion (direct effect = −0.21), and was significantly associated with emotional exhaustion via reappraisal (indirect effect = −0.02) and suppression (indirect effect = −0.05). The total effects of trust among colleagues on teaching satisfaction and emotional exhaustion were 0.44 and −0.28, respectively.

## 4. Discussion

Based on an adjusted JD-R model that considers the mediation of personal resources [2,48], this study examined the relationships between characteristics of teachers’ work environment, teachers’ emotion regulation, and their well-being. To be specific, we hypothesized that the emotional job demands of teaching, as a particular type of job demand, would be detrimental to teachers’ well-being, whereas trust in colleagues, as a type of job resource at the organizational level, would be beneficial to teachers’ well-being. In addition, we hypothesized that the emotion regulation strategies adopted by teachers, acting as personal coping mechanisms [50,51], would mediate the relationships between job characteristics and teacher well-being. Specifically, we hypothesized that reappraisal strategies would play a more favorable role. Our results supported all of the hypotheses.

### 4.1. Contrasting Effects of Emotional Job Demands and Trust in Colleagues

As Bakker and Demerouti concluded, job demands can lead to health problems such as fatigue, energy depletion, and work strain, whereas job resources can be motivational and are associated with high engagement, better wellness, and excellent performance [1]. Our results indicated that emotional job demands and trust in colleagues may associate with teacher well-being differently, demonstrating the detrimental role of job demands and the beneficial role of job resources. These findings are generally consistent with those of previous studies that applied the JD-R model in other work contexts (e.g., [3,4,5,29,43]). They provide further evidence of the applicability of the JD-R model in school settings.

Specifically, this study focused on two characteristics of teachers’ work environment that have been underexplored in the existing literature: the emotional job demands of teaching and trust in colleagues. As some researchers have pointed out, policy and research on teaching and teacher development has usually ignored or minimized the emotional significance of teachers’ work [23,30]. However, echoing Yin’s argument, the results of this study indicated that teachers should be seen as emotional workers, who need to be highly sensitive to the demands that their work makes on their emotions and well-being [61]. Given the high mean score of emotional job demands reported in this study, further attention should be paid to the adverse effects of the emotional demands faced by teachers.

The beneficial role of trust in colleagues is supported by the evidence of its desirable relationship with teacher well-being and emotion regulation strategies (i.e., it increases reappraisal and decreases suppression). Moreover, trust in colleagues is positively related to teaching satisfaction and less emotional exhaustion via the indirect effect of, respectively, reappraisal or suppression. A possible reason for the overwhelmingly beneficial role played by trust in colleagues may be that trust helps to create a work environment that emphasizes authenticity [66], which further encourages teachers to interpret their colleagues’ intentions and behavior as arising from good will [67]. In addition, with regard to the creation of high-quality work environments, trust in colleagues may serve as a determinant and an indicator that teachers’ needs of safety, belongingness, and self-esteem have been satisfied to a certain degree [68]. The fulfillment of these needs further contributes to teachers’ subjective well-being and positive health (i.e., less emotional exhaustion and more satisfaction) [69,70]. Together with the previous findings about the positive effects of trust on teachers’ efficacy, commitment, and well-being indicators [44,45,46,47], our results argue that the establishment of a trusting environment in schools is crucial for the development of teachers’ resilience and professional competence.

### 4.2. Mediation of Teachers’ Emotion Regulation

This study contributes to the literature by integrating personal resources into the JD-R model as a mediating process. Even though trust in colleagues was positively rather than negatively related to reappraisal as supposed, the results of the SEM analyses and Sobel tests supports the partial mediation effect of emotion regulation on the relationships between job characteristics and teacher well-being. Along with recent research on the JD-R model [2,48], our findings indicate that the inclusion of personal resources increases the explanatory power of the JD-R model because it helps to clarify the interaction mechanisms of job characteristics and teachers’ well-being.

The negative relationship between trust in colleagues and suppression confirms our hypothesis that trustful relationships will encourage teachers to be authentic, or to be themselves without any expressive suppression. In contrast, the unexpected positive relationship between trust in colleagues and reappraisal can be explained by Dirks and Ferrin who argued that a trustful environment may affect individuals’ expectations and interpretations of their colleagues’ behavior [67]. Trust may enable a teacher to believe that their colleagues are willing to do good for him or her, and thus he or she will reinterpret the poor behavior of others as only accidental rather than as a conscious betrayal (i.e., reappraisal). Hence, reappraisal may be more likely a regulation in good faith.

Moreover, the results of this study support Lazarus’s claim that coping strategies mediate emotional outcomes [50]. Although a few studies have examined how coping strategies mediate the relationships between job demands and employees’ psychological outcomes [43,54], this study takes a further step by showing the different mediating effects of two emotion regulation strategies. Specifically, the study shows that via the indirect effect of reappraisal, emotional job demands are positively related to teaching satisfaction and negatively associated with emotional exhaustion; in contrast, via the indirect effect of suppression, emotional job demands are negatively related to teaching satisfaction and positively associated with emotional exhaustion. These findings suggest that reappraisal strategies may be more favorable than suppression strategies in mediating the relations between job characteristics and teacher well-being. Therefore, teachers who tend to use more reappraisal strategies may be psychologically healthier than those who tend to adopt more suppression strategies in schools. With these findings, this study lends support to the argument that specific strategies for regulating emotions may be more adaptive and effective for teachers [23,27].

### 4.3. Implications for Practice

Schools are complex emotional arenas and teaching is a form of emotional labor. However, although much attention has been paid to the development of teachers’ professional skills or cognitive aspects like knowledge and thinking, the emotional side of teachers’ work has been largely neglected [30]. This study follows Seligman’s approaches to research on positive health in that it considers factors related to mental health (i.e., trust) as well as mental illness (i.e., EJD) [70], and provides clear evidence about the significance of teachers’ work environment and emotion regulation for their well-being. These results have the following implications for improving the school environment and the health protection of teachers.

First, as teaching is a highly demanding job and teachers must regulate their emotions at work, teachers must be aware of the emotional job demands of teaching. In view of the unfavorable relationships between emotional job demands and teachers’ emotion regulation and well-being, teacher educators and teacher trainers should make the emotional demands of teaching clear in teacher development programs. A comprehensive understanding of the demands of teaching could make in-service teachers and teacher candidates more aware of the influence of teaching on their well-being and effectiveness.

Second, well-being should become a primary focus of organizations and policy makers [71]. The present study demonstrates the importance of a trustful school environment for fulfilling teachers’ needs and protecting their subjective well-being [68,69]. It is advisable that school administrators put more effort into developing trusting relationships among colleagues and cultivating a climate of authenticity in schools. School administrators should provide more opportunities for facilitating collaboration among colleagues, because a sense of trust derives from intense, long-term interpersonal interactions [40]. Meanwhile, school administrators should strive to ensure the openness and transparency of policy making and implementation, and school policies must demonstrate an expectation of trustworthy behavior [31].

Third, given the demonstrated significance of emotion regulation for teacher well-being and the different roles of various emotion regulation strategies, knowledge of the nature and strategies of emotion regulation should be strengthened in teacher education and teacher development. Sutton and Harper have provided good examples of teachers’ emotion regulation at five stages from emotional stimuli to emotional expression [26]; Yin also summarized seven specific strategies for teachers to regulate their emotions in schools [27]. These studies could be the foundation for new designs of teacher development programs. Furthermore, teachers have to be reminded of the different functions of various emotion regulation strategies on their well-being and teaching effectiveness. Based on the results of this study, teachers are encouraged to use antecedent-focused strategies (e.g., reappraisal) rather than response-focused strategies (e.g., suppression) in their work.

### 4.4. Suggestions for Further Research

In this study, the trust relationship between colleagues was focused on because it is more directly associated with variables such as supportive environment and job autonomy which are of great concern in the research field of emotional labor. Trust in colleagues may be more relevant to be considered as the resource side of teaching corresponding to the emotional job demands in this study. However, teachers also have to interact with people other than colleagues at school, such as students and parents. It is possible that teachers’ trust in students and parents may help relieve their emotional pressure during teacher-student/parent interactions. Traditionally, the trust relationships between teachers and students and that between teachers and parents mean that teachers tend to believe that students will do their best to complete their studies and parents will provide sufficient support. Meanwhile, students and parents tend to believe that their teachers will do a good job in terms of both teaching quantity and teaching quality [72,73,74,75]. More work should be done to capture the associations between trust in students or parent and teachers’ emotional well-being in future.

In addition, emotional regulation strategies (i.e., reappraisal and suppression) were treated as mediators between the relationships of emotional job demands and teacher well-being in this study. The stress of emotional job demands may trigger teachers’ adoption of certain strategies, which further influence teachers’ well-being [50]. However, it is also possible that emotional job demands may link more strongly to emotional exhaustion and teaching satisfaction if teachers engage more in suppression strategies, while the use of reappraisal may weaken the relationship between emotional job demands and emotional exhaustion and teaching satisfaction. In other words, emotional regulation may also act as moderators. The present paper only provided some insights about the mediation role of emotional regulation strategies. Future studies may contribute to this line of research by examining both mediating and moderating effects of emotional regulation to obtain a more comprehensive understanding of the role played by emotional regulations.

### 4.5. Limitations

Although this study demonstrates the relationships between job characteristics, emotion regulation, and teacher well-being, and offers some implications for practice, some limitations in its design and analysis have to be addressed.

First, all of the data were collected by self-reported measures, and thus our results may suffer from the common-method bias. Even if teachers know their own emotions and feelings best, further research should use more data sources (e.g., observation and other stakeholders’ accounts) to get more objective data. In addition, the cross-sectional design of the study makes it difficult to claim any causal relationships, although the hypothesized model tested here is based on well-established theories. Future longitudinal or experimental designs will help to clarify the causal relationships between constructs.

Second, it should be noted that all of the participants were from primary schools in Hong Kong, and the results may suffer from the nested data structure of the sample. In this study, we considered trust in colleagues as an individual-level, rather than school-level, variable. Therefore, we did not conduct multilevel analysis by clustering teachers within their school of employment. Therefore, the generalizability of the findings should be treated with caution. Future studies are encouraged to use multilevel analysis to examine the relationships of interest across schools.

Third, all the questionnaires used in this paper were job-specific with the exception of ERQ. Even though we had made some adjustment by adding “in teaching” to each ERQ items when preparing our Chinese version questionnaires, this instrument still needs improvement. Researchers are suggested to use some more job-specific measures regarding teachers’ emotion regulation in their future work.

Fourth, the goodness-of-fit indices of the model tested in this study were near to the thresholds. It implies that there is a risk that the CFA, TLI and RMSEA may fail to reject clearly mis-specified models using the classical cut-off criteria [76]. In future, more work could be done in different contexts to verify the results we have obtained in this current study.

## 5. Conclusions

Using an adjusted JD-R model that integrates the mediation of personal resources, this study examined the relationships between work environment and teacher well-being, and in particular, the mediating role of teachers’ emotion regulation. The results revealed the contrasting interaction patterns of emotional job demands and trust in colleagues with teacher well-being. Moreover, the differences in the mediation effects of the two emotion regulation strategies were highlighted. These findings indicate that to protect teachers’ health, teachers should be fully aware of the characteristics of the teaching profession and the nature of emotion regulation, and need to work in an environment of trust in the school. This study provides insights for improving teacher development and building high-quality work environments through the development of a trustful climate in schools.

## Figures and Tables

**Table 1:** Means, standard deviations, bivariate correlations, and reliability measures of all of the variables (*N* = 1115).

Variables	Mean	SD	1	2	3	4	5	6
1. Emotional job demands	3.94	0.51	(0.69)					
2. Trust among colleagues	3.61	0.62	0.01	(0.88)				
3. Reappraisal	3.71	0.45	0.34 **	0.10 **	(0.79)			
4. Suppression	3.07	0.66	0.25 **	−0.11 **	0.32 **	(0.72)		
5. Emotional Exhaustion	3.61	0.87	0.40 **	−0.26 **	0.09 **	0.36 **	(0.91)	
6. Teaching Satisfaction	3.49	0.71	−0.04	0.39 **	0.16 **	−0.13 **	−0.49 **	(0.89)

*Note*: ** *p* < 0.01; SD = Standard Deviation; Cronbach’s α in parentheses along the diagonal.

**Table 2:** Standard direct, indirect, and total effects.

IV	DV	Type of Effects		Size of the Effects	Sobel Test	Total Effect
EJD	EE	Direct Effect		0.49 ***	-	0.51
		Indirect Effect	Reappraisal	−0.11	−4.67 ***	
			Suppression	0.12	5.71 ***	
	TS	Direct Effect		−0.12 **	-	−0.07
		Indirect Effect	Reappraisal	0.12	4.88 ***	
			Suppression	−0.07	−3.75 ***	
Trust	EE	Direct Effect		−0.21 ***	-	−0.28
		Indirect Effect	Reappraisal	−0.02	−2.78 **	
			Suppression	−0.05	−3.58 ***	
	TS	Direct Effect		0.39 ***	-	0.44
		Indirect Effect	Reappraisal	0.03	2.82 **	
			Suppression	0.03	2.9 **	

*Note*: IV = Independent Variables, DV = Dependent Variables; EJD = Emotional Job Demands, Trust = Trust among Colleagues, TS = Teaching Satisfaction, and EE = Emotional Exhaustion; ** *p* < 0.01, *** *p* < 0.001; although the effects are standard effects, the results of Sobel test were calculated using the raw (unstandardized) regression coefficients [65].
